# The complete mitochondrial genome of click beetle *Agrypnus* sp. (Coleoptera: Elateridae) and phylogenetic analysis

**DOI:** 10.1080/23802359.2019.1673681

**Published:** 2019-10-04

**Authors:** Yimin Du, Xinjun Liu, Yunfei Long, Shuyuan Cheng, Balian Zhong

**Affiliations:** a School of Life Sciences, Gannan Normal University, Ganzhou, China;; b National Navel Orange Engineering and Technology Research Center, Ganzhou, China

**Keywords:** Elateridae, mitochondrial genome, *Agrypnus* sp., phylogenetic analysis

## Abstract

Larvae of click beetles are the most important soil-dwelling agricultural pests and are abundant throughout the world. *Agrypnus* sp. belongs to the subfamily Agrypninae of Elateridae. Here, we sequenced and annotated the complete mitochondrial genome (mitogenome) of *Agrypnus* sp., the new representative of the mitogenome of the subfamily. This mitogenome was 16,056 bp long and encoded 13 protein-coding genes (PCGs), 22 transfer RNA genes (tRNAs), and two ribosomal RNA unit genes (rRNAs). Gene order was conserved and identical to most other previously sequenced Elateridae. The nucleotide composition biases towards A and T, which together made up 70.8% of the entirety. Phylogenetic analysis based on 13 PCGs sequences showed that *Agrypnus* sp. got together with the same subfamily species *Pyrophorus divergens*, *Pyrearinus termitilluminans*, *Ignelater luminosus*, and *Hapsodrilus ignifer* with high support value.

Click beetles (Coleoptera: Elateridae) are the largest family of Elateroidea and include approximately 9000 species. Species in this group often exhibit a unique and well-known startling defense mechanism called ‘clicking’ (Sagegami-Oba et al. 2007). Wireworms, the larvae of click beetles, are the most important soil-dwelling agricultural pests worldwide and can damage potato, carrot, cereals, sugar beet, sugarcane, and soft fruits (Toth et al. 2003; Barsics et al. 2013).

The specimens of *Agrypnus* sp. used for this study were collected from Ledong County, Hainan Province, China (18°46′N, 108°53′E, May 2017) and were stored in Entomological Museum of Gannan Normal University (Accession number GNU-AGRY041). After morphological identification, total genomic DNA was extracted from tissues using DNeasy DNA Extraction kit (Qiagen, Hilden, Germany ). Mitogenome sequence was generated using Illumina HiSeq 2500 Sequencing System. In total, 6.5 G raw reads were obtained, quality-trimmed, and assembled using MITObim v 1.7 (Hahn et al. 2013). By comparison with the homologous sequences of other Elateridae species from GenBank, the mitogenome of *Agrypnus* sp. was annotated using software GENEIOUS R8 (Biomatters Ltd., Auckland, New Zealand).

The complete mitogenome of *Agrypnus* sp. is 16,056 bp (Genbank accession, MN370897). It contains 13 protein-coding genes (PCGs), 22 tRNA genes, 2 rRNA genes, and 1 non-coding AT-rich region. The nucleotide composition of the mitogenome was biased toward A and T, with 70.8% of A + T content (A 40.2%, T 30.6%, C 18.6%, G 10.6%). Gene order was conserved and identical to that of *Drosophila yakuba* and to most other previously sequenced Elateridae (Amaral et al. 2016; Linard et al. 2016; Wang et al. 2019). Four PCGs (*nad4*, *nad4l*, *nad5*, and *nad1*) were encoded by the minority strand (N-strand) while the other nine were located on the majority strand (J-strand). Most PCGs of *Agrypnus* sp. have the conventional start codons ATN (five ATG, three ATA, and three ATT), with the exception of *nad1* (TTG) and *cox1* (AAC), as the asparagine (AAT or AAC) are proposed to be the start codon for *cox1* in suborder Polyphaga (Sheffield et al. 2008). Except for four genes (*cox2*, *cox3*, *nad5*, and *nad4*) end with the incomplete stop codon T−, all other PCGs terminated with the stop codon TAA or TAG. The 22 tRNA genes vary from 62 bp (*trnC*) to 70 bp (*trnK* and *trnV*). Two rRNA genes (*rrnL* and *rrnS*) locate at *trnL1*/*trnV* and *trnV*/control region, respectively. The lengths of *rrnL* and *rrnS* in *Agrypnus* sp. are 1281 and 736 bp, with the AT contents of 75.3 and 73.5%, respectively.

Phylogenetic tree was constructed using the maximum-likelihood method through raxmlGUI 1.5 (Silvestro and Michalak 2012) based on 13 mitochondrial protein-coding genes sequences. Results showed that the newly sequenced species *Agrypnus* sp. got together with the same subfamily Agrypninae species *Pyrophorus divergens*, *Pyrearinus termitilluminans*, *Ignelater luminosus*, and *Hapsodrilus ignifer* with high support value (Figure 1). In conclusion, the mitogenome of *Agrypnus* sp. is obtained in this study and can provide essential and important DNA molecular data for further phylogenetic and evolutionary analysis of Elateridae.

## Figures and Tables

**Figure 1.**
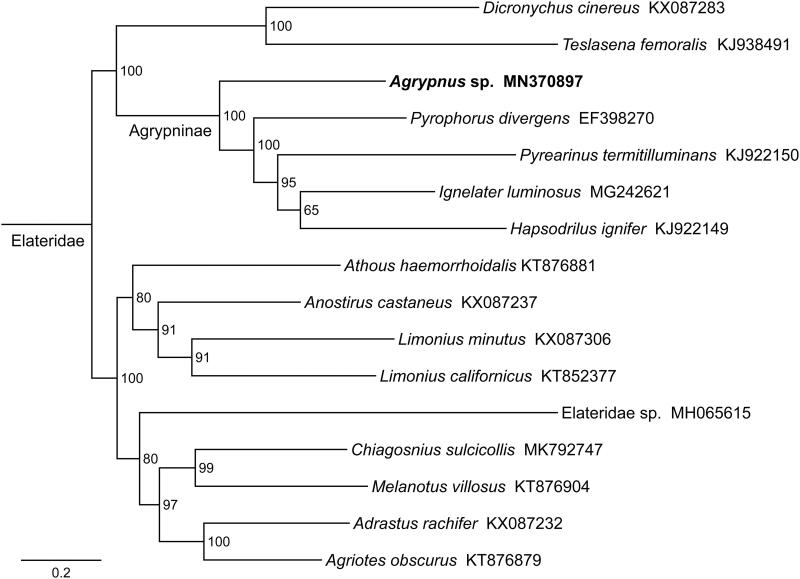
Phylogenetic relationships based on the 13 mitochondrial protein-coding genes sequences inferred from RaxML. Numbers on branches are Bootstrap support values (BS).
